# Melatonin and Testicular Damage in Busulfan Treated Mice

**DOI:** 10.5812/ircmj.14463

**Published:** 2014-02-03

**Authors:** Mehri Mirhoseini, Ghasem Saki, Masoud Hemadi, Ali Khodadadi, Javad Mohammadi Asl

**Affiliations:** 1 Department of Anatomy, School of Medicine, Ahvaz Jundishapur University of Medical Sciences, Ahvaz, IR Iran; 2 Physiology Research Center, School of Medicine, Ahvaz Jundishapur University of Medical Sciences, Ahvaz, IR Iran; 3 Fertility, Infertility and Perinatology Research Center, School of Medicine, Ahvaz Jundishapur University of Medical Sciences, Ahvaz, IR Iran; 4 Cancer, Petroleum and Environmental Pollutants Research Center, Ahvaz Jundishapur University of Medical Sciences, Ahvaz, IR Iran

**Keywords:** Melatonin, Spermatogenesis, Busulfan, Mice, Testis

## Abstract

**Background:**

Advancement in the treatment of various types of cancer has led to greater patient survival. These treatments essentially have toxic effects on different kinds of cells, such as germ cells. Infertility as one of the side effects of cancer treatment has changed the quality of life of young cancer survivors dramatically. Melatonin is an antioxidant with receptors in the reproductive systems.

**Objectives:**

We supposed that melatonin, as an antioxidant, may protect testis against the toxic effects of the drugs.

**Materials and Methods:**

In this experimental study, three groups with seven mice each, were allocated. The control group received normal saline for two months, and the busulfan group received a single dose of 40 mg/kg busulfan intra-peritoneally, and the melatonin group received 20 mg/kg melatonin daily for two months, 45 days after a single dose of busulfan. Next, after decapitation and removal of the testis, tissues were fixed in Bouin's solution and stained by H&E and TUNEL. The sections were evaluated, assessing morphology and spermatogenesis.

**Results:**

In this research, a significant reduction in Johnson’s criteria in the busulfan group (Mean rank = 15.50) was found versus the control group (Mean rank = 45.50), P < 0.001 and in the melatonin group (Mean rank = 45.50) compared to the busulfan group (Mean rank = 15.50), P < 0.001. There was a significant difference between the melatonin and control groups, P < 0.05. In addition, a significant decrease in seminiferous tubule diameter was observed in the busulfan group (763.2 ± 104.41) versus the control group (855.4 ± 52.35), P < 0.01 and melatonin group (834.2 ± 87.26), P < 0.05. Testicular epithelium height was significantly decreased in the busulfan group (Mean rank = 14.60) compared to the control group (Mean rank = 26.40), P < 0.01 and in the busulfan group (Mean rank = 14.95) in comparison with the melatonin group (Mean rank = 26.05), P < 0.01. Also melatonin group (Mean rank = 25.42) showed a significant reduction in epithelium height compared to the control group (Mean rank = 35.58), P < 0.05. Spermatogenesis was impaired in the busulfan group. Although melatonin reduced the rate of apoptosis in the busulfan group, yet it could not remove all apoptotic cells.

**Conclusions:**

This study indicated that melatonin ameliorates the cytotoxic effects of busulfan on germ cells.

## 1. Background

An extensive development in childhood cancer treatment in the recent years has led to a growing population of survivors of childhood malignancies. Therefore, life expectancy has increased (1). Cancer treatments are highly effective, but they cannot specifically target tumoral cells. A major problem in this regard is infertility due to oncological treatments (2). Alkylating agents, which are a kind of cytotoxic chemotherapy drug, can damage the germinal epithelium leading to oligospermia or azoospermia (2). Many attempts have been made to treat infertility of cancer survivors so far. Although semen freezing is a common clinical practice in adults, and fertility rate especially in intra-cytoplasmic sperm injection (ICSI) has greatly improved, this method cannot be applied for young men (3). In fact, there are only Sertoli cells and different types of spermatogonia in immature testicular tissue, which the stem cells are found among them (4).

Busulfan (1, 4-butanediol dimethanesulfonate [myleran]) is a bifunctional alkylating agent and is used for the treatment of various malignant diseases, such as chronic myelogenous leukemia and polycythemia vera (4, 5). Busulfan is a highly toxic agent, which can induce various adverse effects, both acute and chronic, in several biological organs such as hematologic (6), nervous (7) and reproductive organs (8). Using alkylating agents in chemotherapy may induce various cellular mechanisms in germ cells and somatic cells. Transferring the alkyl group(s) to various cellular constituents can cause cytotoxic effects. However, DNA alkylation events may constitute major incitements leading to cell death (9). Simone et al. declared that busulfan kills cells by producing free radicals (10). Antioxidants neutralize both free radicals and the oxidative reactions caused by them (10). Melatonin (MLT), an endogenous indolamine is produced in mammalian pineal glands. Researches during 1993 demonstrated that melatonin functions as a direct free radical scavenger, which can detoxify highly reactive hydroxyl free radicals (OH) *in vitro* (11). Moreover, numerous publications have approved that melatonin and its metabolites can reduce oxidative stress *in vitro* (11). Nowadays researchers believe that melatonin receptors exist in many areas of the brain, the pituitary gland and peripheral tissues such as the reproductive organs (12, 13). So far, several studies have been performed on the effects of melatonin on the reproductive system (14-17). However, reports have shown rather contradictory results of antioxidant activities in male germ cells.

## 2. Objectives

The aim of the present research was to examine melatonin’s ability to improve the quality of spermatogenesis.

## 3. Materials and Methods

### 3.1. Animals

In this study, twenty-one male NMRI (Naval Medical Research Institute) mice (6 – 7 weeks old, 25 – 30 g) were used. The experimental animals were obtained from Ahvaz Jundishapur University of Medical Sciences, Experimental animal Research Center. This study was approved by the ethics committee of Jundishapur University (code; Ajums.rec.1392.125 date; 2013.10.5), and performed according to provided guidelines. Standard laboratory conditions were kept (12 hours dark and 12 hours light cycle, relative humidity of 50 ± 5% and temperature of 22 ± 3°C) one week before the experiment and those conditions were preserved until the end of the experiment. Animal cages were kept clean, and commercial food (pellet) and water were provided ad libitum.

### 3.2. Grouping

Sample size was calculated as two mice per group by the following formula: N = with a power of 0.9 and type II error of 0.1; however, we assigned 7 mice to each group. The test power was 0.896. Mice in Busulfan group received a single dose of 40 mg/kg busulfan intra peritoneally and melatonin group received 20 mg/kg melatonin daily for two months, 45 days after busulfan treatment. In the control group, mice received intra-peritoneal injection of normal saline daily for two months. Busulfan dose was assigned based on previous researches that demonstrated the toxic effect of busulfan on testes (16), and melatonin dose was selected based on previous reports demonstrating its anti-oxidative effect (14).

### 3.3. Organ Removal and Tissue Processing

Animals were killed by decapitation under ether anesthesia, and testes of the animals were removed and fixed in Bouin's solution. The samples were dehydrated and paraffin block were prepared. Next, 5 μm serial sections were prepared, and for histological assessment five slides from each testis were stained with hematoxylin and eosin and TUNEL staining for apoptosis assessment. Samples were analyzed under blindfold conditions.

### 3.4. Histopathology

The seminiferous tubules diameters and the epithelium thicknesses were evaluated by using calibrated linear scale of the Analysis LS Starter software in the 10X eyepiece of Olympus microscope at 40X objective lens. Only circular and near circular tubules were examined. The seminiferous epithelium thickness was calculated by subtracting the lumen diameter from the tubule diameter divided by two (18, 19).

### 3.5. Assessment of Spermatogenesis

Light microscopy was used for the evaluations. Testicular damage and spermatogenesis were graded histopathologically using the Johnson's mean testicular biopsy score (MTBS criteria) (20). Johnson scoring is a common technique to evaluate spermatogenesis. By using a 40× magnification, 100 tubules for each animal were assessed and each tubule was given a grade ranging from 1 to 10. Score one was considered for the tubules with complete inactivity, and score 10 for those with maximum activity (at least five or more spermatozoa in the lumen).

### 3.6. TUNEL Assay (TdT-Mediated dUTP-X Nicked end Labeling)

TUNEL test was performed according to the manufacturer's instructions. After fixation in Bouin's solution, tissues were dehydrated in ethanol (alternatively 70%, 90%, and 100%) and embedded in paraffin. Before the TUNEL staining, testicular sections (5 µm) were rehydrated (xylene 5 minutes; ethanol 100%, 95%, 70%, 2 minutes each) and washed in distilled water. The sections were incubated with proteinase K (20 µg/mL in 10 mM Tris/HCL, PH 7.4-8) at room temperature for 30 minutes and washed with PBS. Sections were re-washed two times with PBS and incubated in a moist chamber with the TUNEL reaction mixture (calf thymus terminal deoxy nucleotidyl transferase, recombinant in *E. coli*, in storage buffer) for 60 minutes at 37 ºC. The sections were incubated in POD after washing two times with PBS (anti fluorescein antibody, FAB fragment from sheep, conjugated with horse-radish peroxidase) in a moist chamber for 10 minutes at 37 ºC. After three PBS washes, DAB detection was performed. Next, sections were washed two times in PBS and were counterstained with hematoxylin. After washing and dehydration, sections were mounted by the entellan mount.

### 3.7. Statistical Analysis

Normal assumption was checked for the tests. In the test, the P value for the diameter was > 0.05, so the data was analyzed using one-way ANOVA followed by Post hoc LSD test and was presented as the mean ± SD. The P value for the Johnson criteria and the height of epithelium was < 0.05, so the data was analyzed using Kruskal-Wallis H. P < 0.05 was considered significant. Data was analyzed using the SPSS software (version 16. SPSS Inc. United States)

## 4. Results

Normal spermatogenesis was seen in the control group, but degenerative changes in the germ cells and loss of spermatogenesis were seen in busulfan treated mice. Melatonin injection followed by busulfan treatment could improve spermatogenesis.

### 4.1. Histopathologic Changes

#### 4.1.1. Evaluation of Spermatogenesis

Control testis showed normal spermatogenesis. Busulfan treatment destroyed testis tissue, and the mean Johnson score (mean rank = 15.50) was decreased significantly in comparison to the control group (mean rank = 45.50), P < 0.001. Melatonin treatment following busulfan injection could nearly repair spermatogenesis, so a significant increase in the score (mean rank = 45.50) was observed compared to the busulfan group (mean rank = 15.50), P < 0.001. Melatonin group showed a significant decrease in the Johnson score compared to the control group (P < 0.05) (Figure 2). As a result, melatonin reduced the damage of germinal cells due to busulfan treatment, but could not repair it completely.

#### 4.1.2. Seminiferous Tubule Morphometry

**Figure 1.**
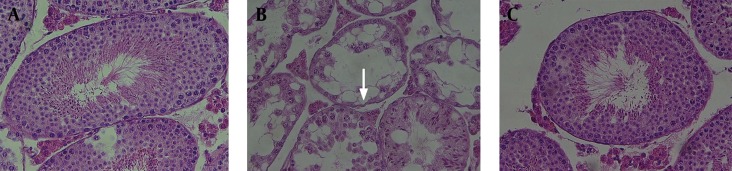
H&E Staining (A) The control group with normal spermatogenesis. (B) Section of testis of a busulfan treated mouse. Germinal layers are disorganized and there are many vacuoles in the tubules (arrow). (C) Cross section of mice testes, which received melatonin following busulfan treatment. GERM Cells have almost improved. H&E Stained. Magnification: ×400.

**Figure 2.**
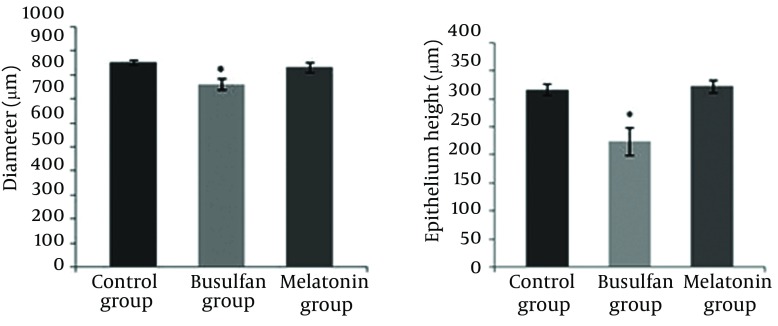
Effect of Busulfan and Protective Effect of Melatonin on Testis Damage Induced by Melatonin Busulfan reduced diameter and thickness of the seminiferous tubules, significantly. Melatonin could significantly repair the injury caused by busulfan. Values are shown as mean ± SD.

In the current study, busulfan disrupted the seminiferous tubules structure, and the germinal cells were degenerated, except for small cells attached to the basement membrane. In addition, as seen in Figure 1 there are many vacuoles in the epithelium indicative of apoptosis in these cells. Melatonin injection could improve the damage (Figure 1). The mean diameter of the seminiferous tubules in the busulfan group (763.2 ± 104.41) indicated a significant decrease in comparison to the control (855.4 ± 52.35), P < 0.01, and melatonin groups (834.2 ± 87.26), P < 0.05, (Table 1). Thickness of the seminiferous tubules in the busulfan group (mean rank = 14.60) decreased significantly in comparison to the control (mean rank = 26.40), P < 0.01, and melatonin groups (mean rank = 26.05), P < 0.01. Also the melatonin group (mean rank = 25.42) showed a significant reduction in epithelium height compared to the control group (mean rank = 35.58), P < 0.05 (Figure 2). 

**Table 1.** Demographic Variables of Seminiferous Tubule Diameters

Group	Mean	SD	SE	P Value	Confidence Interval (Lower, Upper Bound)
**Control**	855.40	52.35	11.70	0.003^a^, 0.708^a^	(830.89, 879.90)
**Busulfan**	763.25	104.41	23.37	0.026, 0.026^a^	(714.38, 812.11)
**Melatonin**	834.25	87.26	19.51	0.708, 0.026^a^	(793.40, 875.09)
**Total**	817.63	91.79	11.85	-	(793.92, 841.34)

^a^ symbols indicate comparison to melatonin and busulfan, respectively. No symbol indicates the control group.

#### 4.1.3. Evaluation of Germ Cell Apoptosis

**Figure 3.**
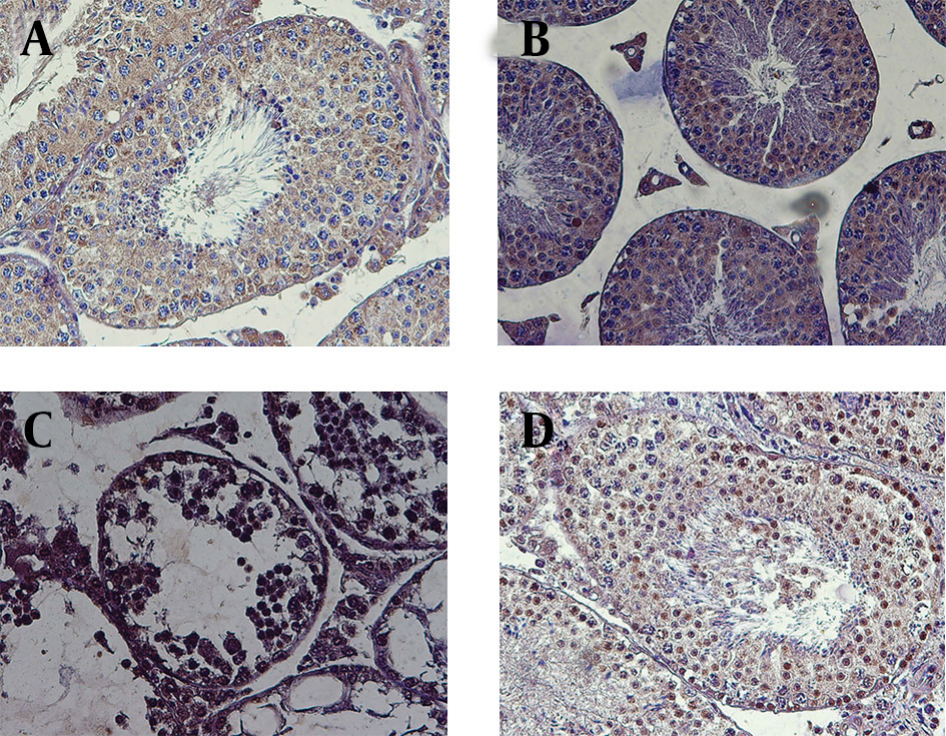
Cross Sections of TUNEL Stained Testes A; Negative control (all nucleuses are blue), B; Control group, which did not receive any drug. There are a few TUNEL positive cells. C; The group, which received busulfan. Most cells were TUNEL Positive. D; Melatonin was injected to mice after receiving busulfan. There were both apoptotic and normal cells. Note that apoptotic cells are indicated by brown nuclei. Magnification: 400X.

TUNEL staining was performed to detect apoptotic cells in the testis tissue (Figure 3). There were only a few TUNEL-positive cells in the control group. Germ cells degeneration in the busulfan group was observed. In addition, TUNEL-positive germ cells also considerably increased in this group. Although melatonin reduced the destructive effects of busulfan on the testis, it could not significantly reduce the number of apoptotic germ cells in mice treated with busulfan.

## 5. Discussion

In this study, the germinal epithelium of male rats was destroyed by a single dose of busulfan, and it was shown that melatonin treatment could repair these defects. The fractionated dose of busulfan was determined based on a previous research (21). We observed the destructive action of busulfan on germ cells as shown in Figure 1. Udagawa et al. indicated that busulfan treatment induces damage to spermatogenesis while recovery from such damage may be difficult (5). Many vacuoles were seen in the germinal epithelium in busulfan group. Vacuoles are considered as an apoptotic sign due to germ cell loss and detachment (22, 23). Apoptosis is a form of cell death, characterized by morphological and biochemical changes such as DNA fragmentation, formation of apoptotic bodies, and little inflammatory response (24).

Testicular apoptosis is found during normal spermatogenesis in mammals and is believed to be essential for the preservation of the correct ratio between sertoli cells and gametes (25). Germ cell apoptosis can also be significantly motivated by many pathological conditions such as heat stress, exposure to ionizing radiation, toxic substances, hormonal depletion, and loss of stem cell factor (SCF) (26). Increase in apoptotic rate in the busulfan group was confirmed by the TUNEL test compared to other groups. Apoptotic effects of busulfan were shown by earlier studies to cause a loss of A1 spermatogonia and primary spermatocytes (16).

Many efforts have been made to improve fertility after cytotoxic therapy; nearly all focused on hormonal treatment (27). Melatonin repaired the testis tissue in this study, and spermatogenesis was resumed after melatonin therapy as seen in Figure 1. Melatonin is synthesized by the pineal gland and is assumed to have free radical scavenger properties (11). Numerous studies have demonstrated the protective action of melatonin and their metabolites against oxidative and nitrosative changes of DNA (11, 28, 29). These modifications are due to many endogenous and exogenous free radical producing processes (11). Melatonin was found to be a protectant against streptozotocin toxicity in diabetic rats (30) and after radiotherapy (31). In this research, although melatonin could reduce the rate of apoptosis in the busulfan group, many apoptotic cells were present after treatment. In agreement with our research, Hemadi et al. dedicated that melatonin can significantly reduce apoptosis, while inducing proliferation of the cells (14). Similarly Mohamad Ghasemi et al. reported reduced apoptosis following melatonin reception (17). These results are probably due to antioxidant properties of melatonin.

Evaluation of antioxidative status in the presence of melatonin is necessary to prove it. Considering that the germinal cells have receptors for melatonin, direct impact on the cells cannot be ruled out. On the contrary, Chen et al. (32) showed that melatonin induces apoptosis in germ cells of male mouse. In this case and some other studies such as that of Succu, melatonin was introduced as a time and dose dependent hormone (33). It has been shown that the prevalence of spermatogonial and spermatid apoptosis in Chinese men is more than Caucasian men (24). It appears that racial differences in the inherent sensitivity of germ cells to programmed cell death can be a factor for this difference. However, the mechanisms by which melatonin effect germ cells remain unknown. Finally, this study showed that melatonin improves the cytotoxic effect of busulfan on germ cells. Thus, it may be used for the protection of germinal layer defects caused by radiotherapy or chemotherapy in human. Obviously, more research is required to generalize these results to humans.
